# Operative Behandlung des Torticollis muscularis congenitus

**DOI:** 10.1007/s00064-023-00805-x

**Published:** 2023-04-20

**Authors:** Milena M. Ploeger, Christoph Trillhaase, Charlotte Rommelspacher, Rahel Bornemann, Robert Ossendorf, Richard Placzek

**Affiliations:** Klinik für Orthopädie und Unfallchirurgie, Universitätsklinikum Bonn, Venusberg-Campus 1, 53127 Bonn, Deutschland

**Keywords:** Torticollis muscularis congenitus, Triterminale Tenotomie, Spiegel-Orthese, Schiefhals, Torticollis-Therapie, Congenital muscular torticollis, Tripolar tenotomy, Orthotic devices, Torticollis therapy

## Abstract

**Operationsziel:**

Operative Korrektur des Torticollis muscularis congenitus (TMC) mit triterminaler Tenotomie des M. sternocleidomastoideus (SCM) sowie postoperativer Behandlung in der Spiegel-Orthese.

**Indikationen:**

Muskulär bedingter Torticollis im Rahmen einer Kontraktur des SCM, nach Versagen der konservativen Therapie ab dem 2. Lebensjahr.

**Kontraindikationen:**

Torticollis im Rahmen einer ossären Anomalie oder aufgrund anderer muskulärer Kontrakturen

**Operationstechnik:**

Durchtrennung des M. sternocleidomastoideus (SCM) am Ursprung und Ansatz (klavikulär und sternal) mit Resektion der Sehne von ca. 1 cm im Bereich des Ansatzes.

**Weiterbehandlung:**

Postoperative Anlage der Spiegel-Orthese. Tragen der Orthese 24 h/Tag für die ersten 6 Wochen postoperativ, danach Tragen der Orthese 12 h/Tag für weitere 6 Wochen.

**Ergebnisse:**

Operiert wurden *n* = 13 Patienten mit TMC. Das Follow-up betrug im Durchschnitt 25,7 Monate, bei 1 Patienten kam es nach 3 Jahren zu einem Rezidiv. Weder intra- noch postoperative Komplikationen wurden beobachtet.

## Abstract (en)

**Objective:**

Surgical treatment of congenital muscular torticollis with tripolar release of the sternocleidomastoid muscle, followed by modified postoperative treatment with a special orthosis.

**Indications:**

Muscular torticollis due to contracture of the sternocleidomastoid muscle; failure of conservative therapy.

**Contraindications:**

Torticollis due to bony anomaly or other muscular contractures.

**Surgical technique:**

Tenotomy of the sternocleidomastoid muscle occipitally and resection of at least 1 cm of the tendon at the sternal and clavicular origin.

**Postoperative management:**

Orthosis must be worn for 6 weeks 24 h/day, then for another 6 weeks 12 h/day.

**Results:**

A total of 13 patients were treated with tripolar release of the sternocleidomastoid muscle and modified postoperative management. Average follow-up time was 25.7 months. One patient presented with recurrence after 3 years. No intra- or postoperative complications were observed.

## Vorbemerkungen

Der Torticollis muscularis congenitus (TMC) gilt neben der kongenitalen Hüftdysplasie und dem Klumpfuß als dritthäufigste angeborene muskuloskeletale Deformität in der Kinderorthopädie [20]. Die Inzidenz unter den Neugeborenen wird in der Literatur mit einer Spanne von 0,3–2 % angegeben [17, 23]. Der angeborene, muskuläre Schiefhals wird durch eine strukturelle, einseitige Verkürzung des M. sternocleidomastoideus (SCM) mit resultierender Kopf-Hals-Fehlhaltung verursacht [15, 24]. Abzugrenzen von Erkrankungen mit einem ähnlichen Krankheitsbild, liegt beim Torticollis per definitionem eine Seitenneigung des Kopfes zur betroffenen Seite und eine gleichzeitige Rotation des Halses zur Gegenseite vor [25].

Als Ursache des TMC wurde lange ein mögliches Geburtstrauma angesehen [8]. Die aktuell am weitesten verbreitete Theorie zur Entstehung des TMC beschreibt ein sekundäres Kompartmentsyndrom nach intrauteriner Fehllage oder perinatalem Geburtstrauma, welches durch Ischämie zur einer vermehrten Fibrosierung der Muskulatur führen kann [24, 26].

Weitere Ursachen des angeborenen Schiefhalses sind zudem knöcherne Deformitäten (Halbwirbelbildungen, Klippel-Feil-Syndrom) oder neurologische Ursachen, die es bei der Therapieindikation zu berücksichtigen gilt [10].

Der im Wachstum aufgetretene Torticollis sollte umfassend interdisziplinär abgeklärt werden, um weitere Ursachen (entzündliche Genese, okulär, neurogen) auszuschließen.

Eine frühe, erfolgreiche Therapie des Schiefhalses ist von eminenter Notwendigkeit, da ein unbehandelter TMC in der Wachstumsphase des Kindes eine irreversible Gesichtsskoliose und eine Störung des Körperschemas zur Folge haben kann [5, 22].

Die primäre Therapie des TMC ist konservativ mit intensiver physiotherapeutischer Behandlung [18]. In der Literatur gibt es keinen klaren Konsens, wie lange die konservative Therapie durchgeführt werden sollte. Einige Autoren geben mindestens 6 bis 12 Monate an, andere bis Abschluss des 1. Lebensjahres [5, 14]. Ein klares Kriterium zur operativen Therapie ist das Auftreten einer Gesichtsskoliose, die sich nach Abschluss des 4. Lebensjahres trotz operativer Therapie des TMC nur noch selten zurückbildet [5, 21]. Bezüglich des operativen Prozedere werden in der Literatur verschiedene Operationstechniken beschrieben. Einige Autoren favorisieren eine unilokuläre, distale Tenotomie beim jungen Patienten [13]. Teilweise wird diese Tenotomie mittels zusätzlicher Durchtrennung der Ursprungssehne erweitert (biterminale Tenotomie) [9, 27]. Eine weitere Option liegt in der Kombination der biterminalen Tenotomie mit zusätzlicher Z‑Plastik im distalen Muskelbereich [9]. Zudem wurden verschiedene endoskopische Verfahren beschrieben [3, 19].

Eine weit verbreitete Nachbehandlungsoption besteht in der kontinuierlichen Anlage eines Thorax-Diadem-Gipses für 6 Wochen postoperativ [7, 12]. Einige Autoren empfehlen, eine Kopfhalftertraktion für 2 bis 4 Wochen und danach eine Zervikalstütze für 3 bis 4 Monate anzulegen [9].

Das Rezidivrisiko nach operativer Therapie variiert in der Literatur deutlich (5,5–26,5 %) [4, 6]. Uneinigkeit besteht jedoch darin, inwiefern Operationstechnik und Alter des Patienten zum Zeitpunkt der Operation einen Einfluss auf das Rezidivrisiko haben [6, 11, 16]. In der Literatur zeigt sich eine Tendenz, dass unilokuläre Tenotomien ein erhöhtes Rezidivrisiko im Vergleich zu biterminalen Tenotomien haben [4].

Nach unserem neuartigen Behandlungsschema erfolgt initial obligat eine konservative Therapie des Kindes bis zum 2. Lebensjahr mittels intensiver Physiotherapie. Wir empfehlen ausdrücklich keine Manipulationen im Rahmen einer osteopathischen Therapie, denn auch wenn neuere Studien einen Zugewinn für den zusätzlichen Einsatz von Chiropraktik/Manualtherapie gegenüber alleiniger Physiotherapie beschreiben, gilt es, schwere Nebenwirkungen bis hin zur Asphyxie zu berücksichtigen [1, 2]. Bei Versagen der konservativen Therapie führen wir unabhängig des Patientenalters direkt eine triterminale Tenotomie des SCM mit Absetzen der mastoidalen, sternalen und klavikulären Sehne durch, um das mögliche Rezidivrisiko zu minimieren. Anschließend wird intraoperativ die Kopf-Hals-Achse manuell korrigiert. Direkt im Anschluss erfolgt im Operationssaal die Anlage einer präoperativ individuell angefertigten sog. Spiegel-Orthese. Diese spezielle *Custom-made*-Orthese soll dabei eine – „quasi gespiegelte“ – Überkorrektur der Ausgangsfehlstellung der Kopf-Hals-Achse gewährleisten. Die Spiegelorthese wird in den ersten 6 Wochen postoperativ 24 h/Tag getragen, anschließend für weitere 6 Wochen zur Nacht (ca. 12 h/Tag). Das Nachbehandlungskonzept wird durch regelmäßige Physiotherapie – beginnend ab dem 1. postoperativen Tag – für weitere 6 Monate komplettiert.

## Operationsprinzip und -ziel

Die Durchtrennung des SCM erfolgt sowohl am Ursprung als auch am Ansatz der Sehne im Rahmen einer triterminalen Tenotomie mit zusätzlicher Resektion der Sehne von ca. 1 cm im Bereich des klavikulären und sternalen Ansatzes. Zur Rezidivprophylaxe erfolgt direkt im Anschluss die Nachbehandlung durch das Anlegen einer sog. Spiegel-Orthese für insgesamt 12 Wochen postoperativ (1. bis 6. Woche 24 h/Tag, 7. bis 12. Woche 12 h/Tag)

## Vorteile

- Zwei kosmetisch unauffällige Hautschnitte von ca. 1–3 cm Länge je nach Größe/Alter des Patienten
- Komplikationsarme Operationstechnik
- Im Vergleich zum Diadem-Gips vereinfachtes postoperatives Behandlungsschema durch Tragen der Orthese
- Beginn der Physiotherapie ab dem ersten postoperativen Tag
- Operationstechnik mit postoperativer Orthesenbehandlung ab dem 2. Lebensjahr möglich

## Nachteile

- Potenzielles Verletzungsrisiko wichtiger neurovaskulärer Strukturen im Operationsgebiet
- Korrektur nur bei muskulär bedingtem Torticollis
- Für den Therapieerfolg und zur Vermeidung von Rezidiven konsequentes Tragen der Orthese für insgesamt 12 Wochen postoperativ notwendig

## Indikationen

- Muskulär bedingter Torticollis im Rahmen einer Kontraktur des SCM
- Nach Versagen der konservativen Therapie ab dem 2. Lebensjahr
- Beginnende Gesichtsskoliose

## Kontraindikationen

- Torticollis im Rahmen einer ossären Anomalie oder Fehlbildung (z. B. Klippel-Feil-Syndrom)
- Torticollis aufgrund anderer muskulärer Kontrakturen (Dystonie, Torticollis spasmoides) oder im Rahmen einer entzündlichen Genese (z. B. Grisel-Syndrom)

## Patientenaufklärung

- Allgemeine Operationsrisiken (Infektion, Thrombose, Embolie, Gefäß- oder Nervenschäden, Nachblutung, Wundheilungsstörungen u. a.)
- Gefäßverletzung mit Blutung: *Aa. subclavia, carotis communis et externa, auricularis *und *Vv. subclavia, jugularis interna et externa*
- Nervenverletzung mit Funktionsverlust: *Nn. facialis, vagus, accessorius*
- Persistenz der eingeschränkten ROM des Kopf-Hals-Bereichs
- Rezidivrisiko
- Unter- oder Überkorrektur
- Ästhetisch störende Narbenbildung, ggf. Keloidbildung
- Fehlendes Therapieansprechen insbesondere bei vorhandener Gesichtsskoliose
- Geringes Risiko eines Pneumothorax
- Postoperatives Tragen der Spiegel-Orthese (zunächst 6 Wochen 24 h/Tag, danach 6 Wochen 12 h/Tag)
- Mögliche Druckstellenbildung durch Orthese
- Regelmäßige, postoperative Physiotherapie für mindestens 6 Monate

## Operationsvorbereitungen

- Klinische Untersuchung der Beweglichkeit der Halswirbelsäule (Ante‑/Retroflexion, Seitneigung nach rechts/links, Rotation rechts/links)
- Röntgenaufnahme der Halswirbelsäule in 2 Ebenen zum Ausschluss struktureller Veränderungen (z. B. Klippel-Feil-Syndrom)
- Gegebenenfalls neurologische, ophthalmologische oder HNO-ärztliche Untersuchung zum Ausschluss einer anderen Torticollis-Genese (neurologisch, okulär, vestibulär)
- Gegebenenfalls weitere bildgebende Diagnostik (MRT Hals/obere Thoraxapertur) bei klinisch nicht eindeutiger Diagnosestellung
- Laborchemische Kontrolle der Infektparameter zum Ausschluss eines Infektgeschehens
- Fotodokumentation
- Präoperative Anfertigung der maßangefertigten Spiegel-Orthese (Anfertigungszeit ca. 4 bis 6 Wochen)

## Instrumentarium

- Chirurgische Klemme (beispielsweise Overholt-Klemme)
- Thermokauter
- Gegebenenfalls stumpfe Hauthaken zur Vermeidung von Wundrandnekrosen
- Langenbeck-Haken
- Kleine stumpfe Klemme

## Anästhesie und Lagerung

**Figure 1:**
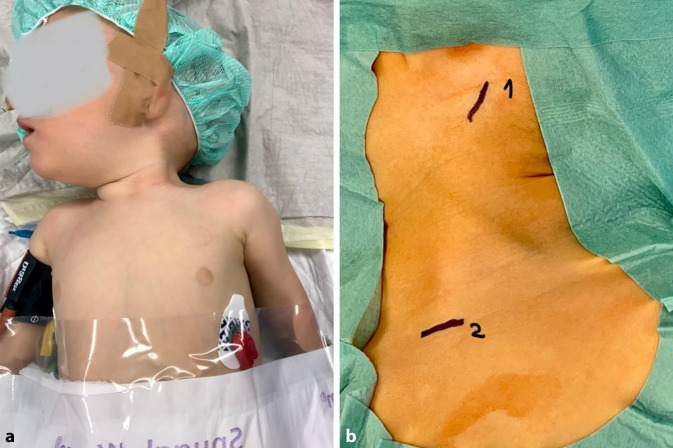

- Vollnarkose
- Rückenlagerung, Lagerung mit Unterpolsterung zwischen den Schulterblättern, sodass die Ansätze des SCM gut zum Vorschein kommen
- Drehung des Kinns zur nicht betroffenen Seite/Aufspannen des betroffenen SCM
- Hochkleben des Ohres der betroffenen Seite, um eine möglichst ansatznahe (am Mastoid) Tenotomie zu ermöglichen (s. Abb. 1)

## Operationstechnik

**Figure 2:**
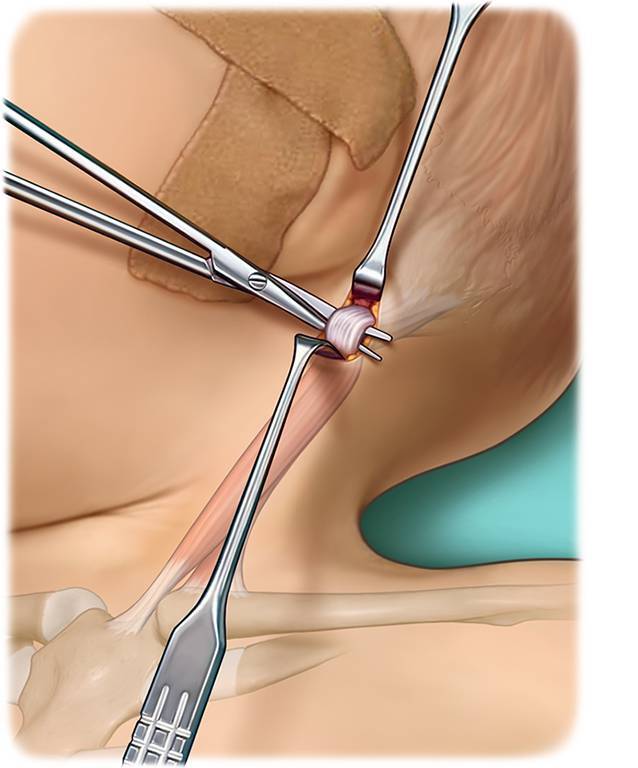

**Figure 3:**
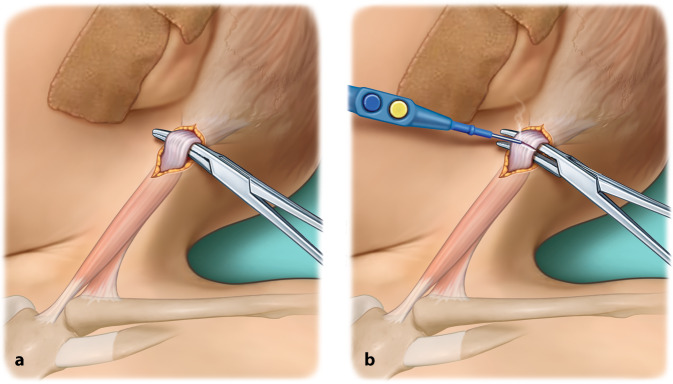

**Figure 4:**
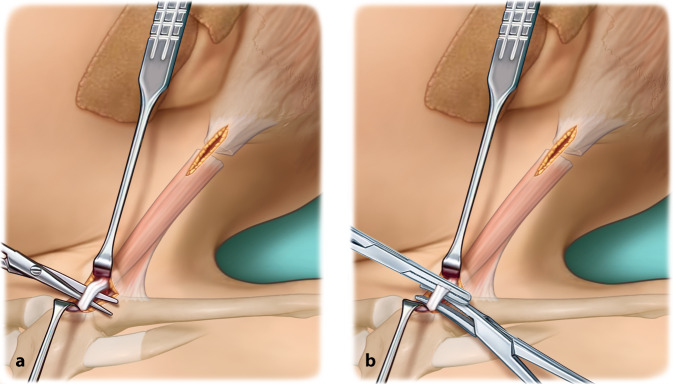

**Figure 5:**
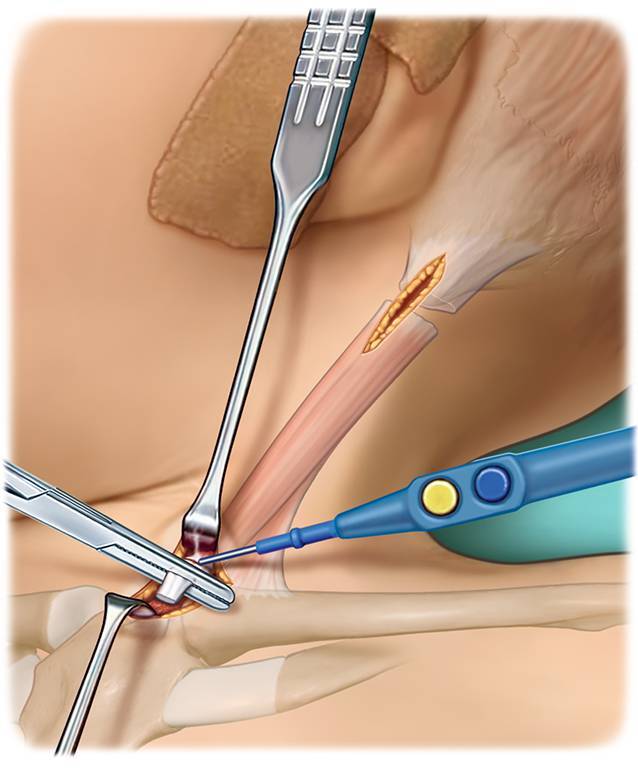

(Abb. 1, 2, 3, 4, 5, 6 und 7)

**Figure 6:**
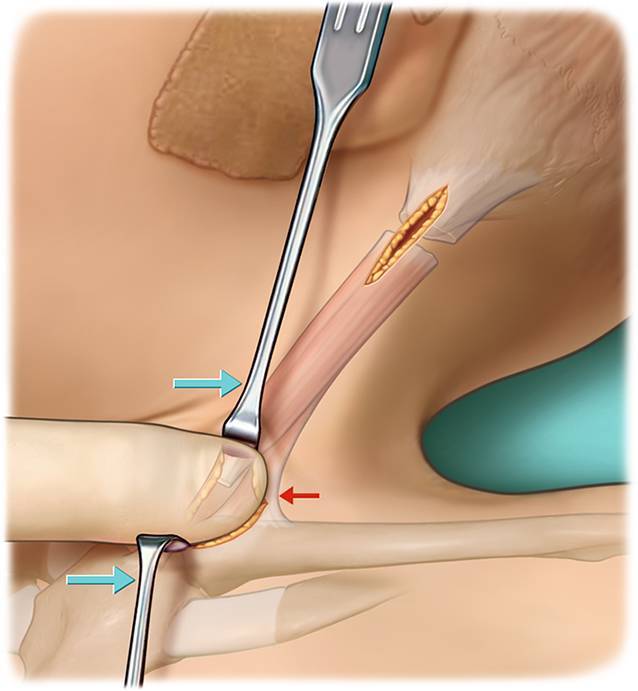

**Figure 7:**
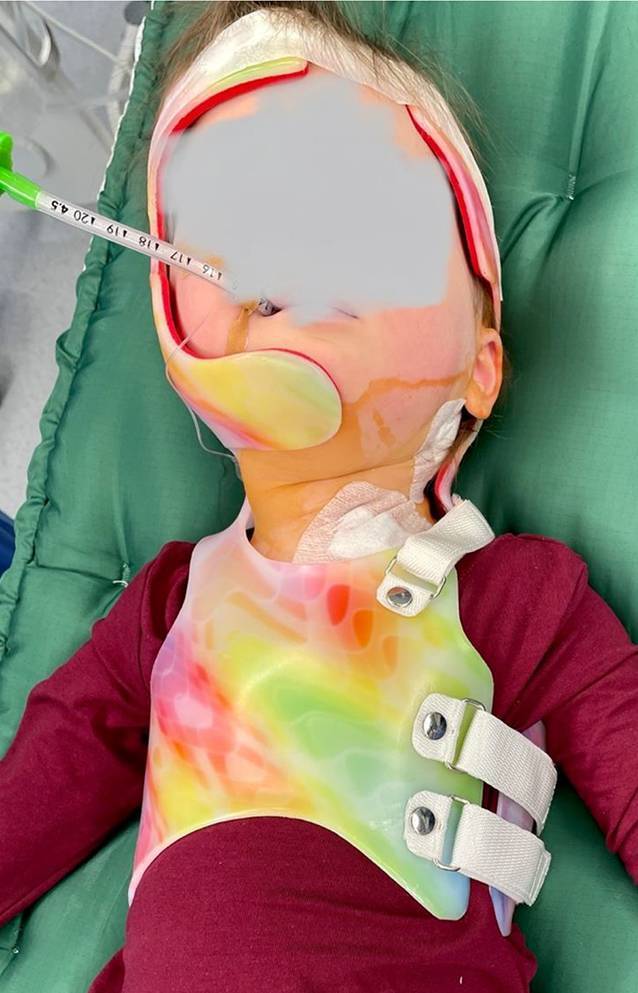

## Besonderheiten

- Resektion von ca. 1 cm im Bereich des sternalen und klavikulären Ansatzes des SCM
- Keine Z‑Plastik der Sehne
- Vereinfachtes postoperatives Prozedere im Vergleich zum Diadem-Gips durch ein mögliches An- und Ablegen der Spiegel-Orthese

## Postoperative Behandlung

- Perioperative Anlage der Spiegel-Orthese
- Tragen der angelegten Orthese 24 h/Tag für die ersten 6 Wochen postoperativ, danach Tragen der Orthese 12 h/Tag insbesondere nachts für weitere 6 Wochen
- Ab dem ersten postoperativen Tag physiotherapeutische Beübung für mindestens 6 Monate
- Ambulante Wiedervorstellung zur klinischen Verlaufskontrolle 6 Wochen postoperativ, 12 Wochen postoperativ sowie nach 6 Monaten.

## Fehler, Gefahren, Komplikationen

- Verletzung des Nn. facialis und accessorius oder begleitender Gefäße (Aa. carotis externa, auricularis, V. jugularis interna) bei proximaler Tenotomie
- Verletzung von Gefäßen (Aa. subclavia und carotis communis, Vv. subclavia und jugularis interna et externa) und Nerven (N. vagus) bei distaler Tenotomie
- Sollte es zu Verletzungen von Blutgefäßen kommen, empfehlen wir zunächst eine Kompression und ggf. Koagulation je nach Gefäß. Je nach Befund (Verletzungen der Aa. subclavia und Aa. carotis communis) zudem Hinzurufen der Gefäßchirurgie indiziert
- Bei Verletzungen des N. facialis kann es postoperativ zur einer möglichen Lähmung der mimischen Muskulatur kommen. Hier empfehlen wir eine neurologische und ggf. neurochirurgische Vorstellung im Verlauf bezüglich einer operativen Rekonstruktion
- Verletzungen des N. accessorius können zu einer Schwäche oder Lähmung des M. trapezius führen. Auch hier empfehlen wir eine postoperative neurologische und neurochirurgische Vorstellung bezüglich einer weiteren Abklärung und ggf. operativen Rekonstruktion
- Potenzielles Risiko für einen iatrogenen, apikalen Pneumothorax bei distaler Tenotomie. Bei Verdacht auf einen Pneumothorax besteht die Indikation zum Röntgen des Thorax und Anlage einer Thoraxdrainage je nach Größe des Pneumothorax
- Rezidive durch unzureichende Exzision der tenotomierten Sehnenanteile und verstärkte Narbenbildung der Sehnenenden mit Indikation zur operativen Revision
- Rezidivrisiko durch fehlende Compliance bezüglich des Nachbehandlungsschemas
- Persistenz der bestehenden Gesichtsskoliose bei zu später operativer Korrektur

## Ergebnisse

Im Zuge einer retrospektiven Studie wurden im Zeitraum von 2015 bis 2020 *n* = 13 Patienten (weiblich *n* = 8) in unserer Abteilung mit therapierefraktärem TMC mittels triterminaler Tenotomie operativ versorgt. Das Durchschnittsalter lag bei 10,4 Jahren (MIN 1,5 Jahre, MAX 40 Jahre). Das Follow-up betrug im Durchschnitt 25,7 Monate (MIN 8 Monate, MAX 52 Monate). Bei allen Patienten bestand ein kongenitaler Torticollis, bei *n* = 3 Patienten wurden im Rahmen der konservativen Therapie neben der Physiotherapie zusätzlich Botox-Infiltrationen durchgeführt. Die mittlere Operationsdauer betrug 37,7 min (MIN 29 min, MAX 42 min).

Weder intra- noch postoperative Komplikation, wie beispielsweise lokale Infektion, Wundheilungsstörungen oder Hämatombildungen, die eine erneute operative Revision zur Folge gehabt hätten, wurden beobachtet. Im Rahmen der postoperativen Behandlung zeigte sich bei *n* = 2 Patienten eine Druckstelle durch die Orthese, sodass diese nachgepolstert wurde.

Keiner der Patienten oder Eltern beklagte postoperativ eine „Wulstbildung“ im Bereich des Sternocleidomastoideus nach erfolgter triterminaler Tenotomie. Klinisch zeigte sich in den Nachkontrollen bei keinem der Patienten ein verändertes Halsrelief, welches ästhetisch störend wirken könnte. Bei einem Patienten kam es nach 3 Jahren zu einem lokalen Rezidiv, welches erneut operativ versorgt wurde.

Die Beweglichkeit der HWS war bei *n* = 12 Patienten im 6 Monats-Follow-up physiologisch mit einer Lateralflexion beidseits von mindestens 45° sowie einer Rotation beidseits von mindestens 80°. Bei einer Patientin zeigte sich nach 6 Monaten noch eine Seitendifferenz bezüglich der Rotation von ca. 30° (Rotation der behandelten Seite 80°, der unbehandelten Seite 50°).

Hinsichtlich der Rezidivneigung zeigt das von uns beschriebene Therapieverfahren im Vergleich zur aktuellen Literatur mit 7,7 % ein geringes Rezidivrisiko [4, 6]. Relevante intraoperative Komplikationen konnten nicht beschrieben werden, insbesondere kam es zu keinen relevanten Gefäß- oder Nervenverletzungen. Trotz triterminaler Tenotomie kam es im Gegensatz zu in der Literatur beschriebenen Fällen zu keinem ästhetisch störenden Befund des Halsreliefs.
